# Maternal Listeriosis Presenting With Suspected Meningitis in Late Pregnancy: A Case Report

**DOI:** 10.7759/cureus.99839

**Published:** 2025-12-22

**Authors:** Nathallie George, Dominique Marshall

**Affiliations:** 1 Obstetrics and Gynaecology, Nottingham University Hospitals NHS Trust, Nottingham, GBR; 2 Obstetrics and Gynaecology, University Hospitals of Leicester NHS Trust, Leicester, GBR

**Keywords:** antibiotic therapy, baterial meningitis, fetal risks, listeria monocytogenes, listeriosis, maternal sepsis

## Abstract

Maternal listeriosis is uncommon but clinically important due to the risk of severe maternal illness and adverse perinatal outcomes. Nonspecific symptoms often overlap with viral and respiratory illnesses, delaying diagnosis. We describe the case of a 28-year-old gravida 4 para 2+1 at 29+5 weeks who presented with fever, frontal headache, photophobia, and generalized myalgias. Significant medical history included asthma, chronic hypertension, chronic hepatitis B and C carriage, and a sickle cell variant. Blood cultures grew *Listeria monocytogenes*. She was treated with intravenous amoxicillin and gentamicin after initial empiric therapy. Despite concern for central nervous system involvement, brain MRI revealed no intracranial pathology. She transitioned to benzylpenicillin for outpatient therapy and achieved complete recovery. A healthy infant was delivered by emergency caesarean section at 39+1 weeks. Maternal listeriosis can closely mimic common viral illnesses. Thus, maintaining a high index of suspicion, especially in pregnant women with nonspecific febrile symptoms, can facilitate timely diagnosis and treatment, which is essential to optimising maternal and neonatal outcomes.

## Introduction

Maternal sepsis remains a major cause of maternal morbidity and mortality worldwide and demands rapid recognition and treatment [1-3]. Physiological changes in pregnancy, tachycardia, tachypnoea, lower systemic vascular resistance, and haemodilution, can mask early signs of sepsis, increasing the risk of deterioration without prompt care [1-3]. Pregnancy-related immunological adaptations, particularly reduced cell-mediated immunity, increase susceptibility to Listeria monocytogenes [4-6]. Infection may be non-invasive and self-limiting (e.g., diarrhoea, vomiting, nausea) or invasive with fulminant multi-organ failure and complications such as bacterial meningitis [6]. Even when maternal symptoms are mild, fetal risks, miscarriage, preterm birth, stillbirth, and neonatal sepsis, remain significant, underscoring the need for rapid diagnosis and appropriate treatment [7,8]. Transmission is mainly foodborne (e.g., unpasteurised dairy, soft cheeses, chilled ready-to-eat foods, refrigerated smoked seafood), and Listeria can multiply at refrigeration temperatures [9].

## Case presentation

A 28-year-old woman (G4P2+1) at 29 weeks + 5 days presented with a one-week history of fever, frontal headache, photophobia, and generalized myalgias unresponsive to regular analgesia. There was no recent travel or sick contacts. Past medical history included asthma, chronic hypertension, chronic hepatitis B and C carriage, and a sickle cell variant. Her pregnancy had been uncomplicated under joint obstetric-hepatobiliary care.

Clinical examination and investigation

Upon review, the patient was febrile (39.5°C) and tachycardic (heart rate (HR) 118 bpm) with a blood pressure reading of 124/61 mmHg, a respiratory rate of 28, and oxygen saturation of 94% on room air. She had nuchal rigidity; however, neurological examination was otherwise normal.

**Figure 1:**
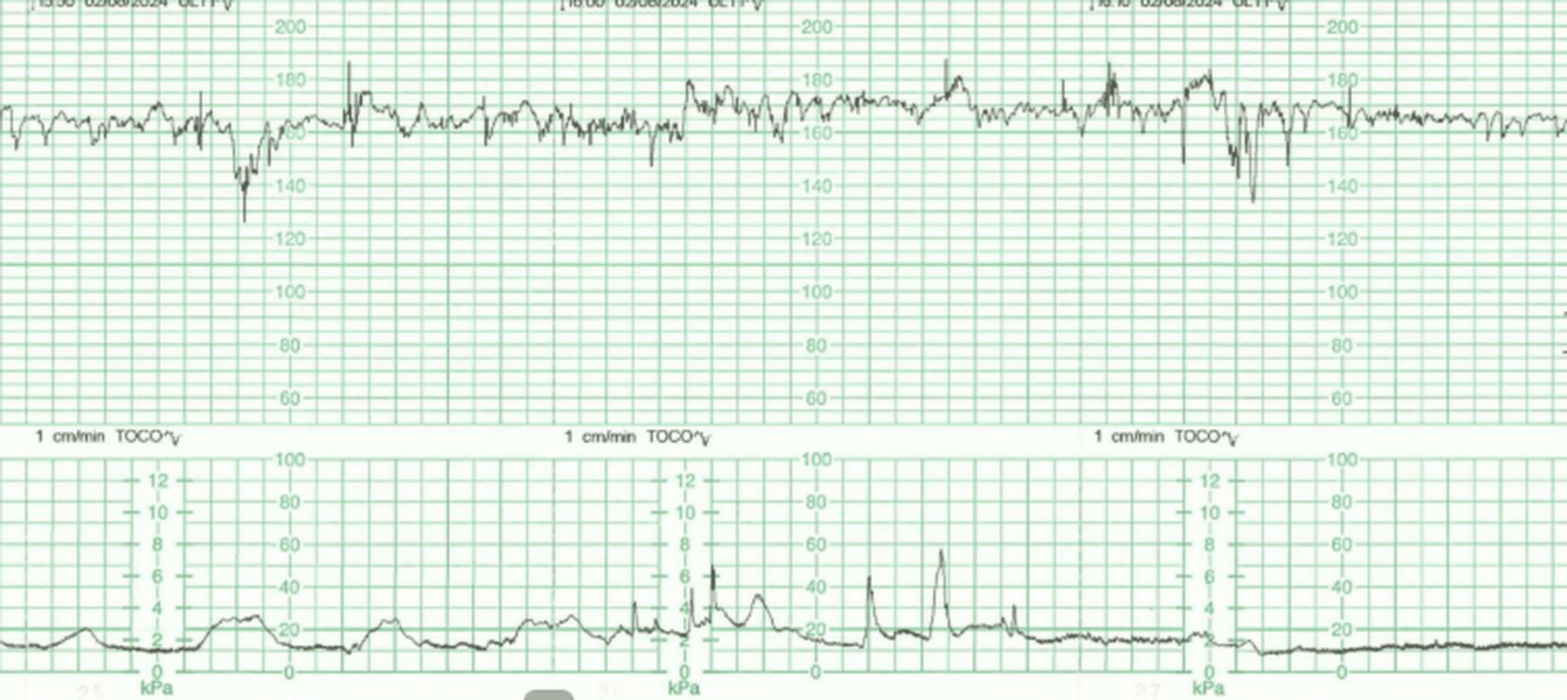
CTG with baseline rate of 170 bpm. CTG: Cardiotocography.

The cardiovascular and respiratory examinations were unremarkable. Her Modified Early Obstetrics Warning System (MEOWS) score, which is used to detect early signs of maternal illness and so reduce maternal morbidity and mortality, was 10 [10]. An antenatal foetal cardiotocography (CTG) was performed using the Dawes-Redman analysis. The criteria for foetal well-being were not met due to a foetal tachycardia of 170 bpm. This persistent baseline rate of 170 bpm lies outside the normal Dawes-Redman range and explained the failure to meet computerized criteria, indicating foetal stress in the context of maternal pyrexia and systemic infection (Figure 1).

She was admitted, and a full septic screen was performed in line with treatment for red-flag sepsis, including viral throat swabs, a vaginal swab, urine for microscopy, culture and sensitivity. The Septic 6 bundle was initiated.

A point-of-care venous blood gas had a lactate of 0.7 mmol/L and haemoglobin of 96 g/L. She was commenced on intravenous Tazocin as per our Trust guidance on “Red Flag Sepsis” within one hour of presentation. She also had simultaneous fluid resuscitation and oxygen therapy along with multidisciplinary input from the anaesthetic consultant, obstetric consultant, and critical care outreach team. This multidisciplinary input was triggered by her MEOWS score, which indicated a high level of clinical concern by reflecting significant abnormalities in her vital signs.

**Figure 2:**
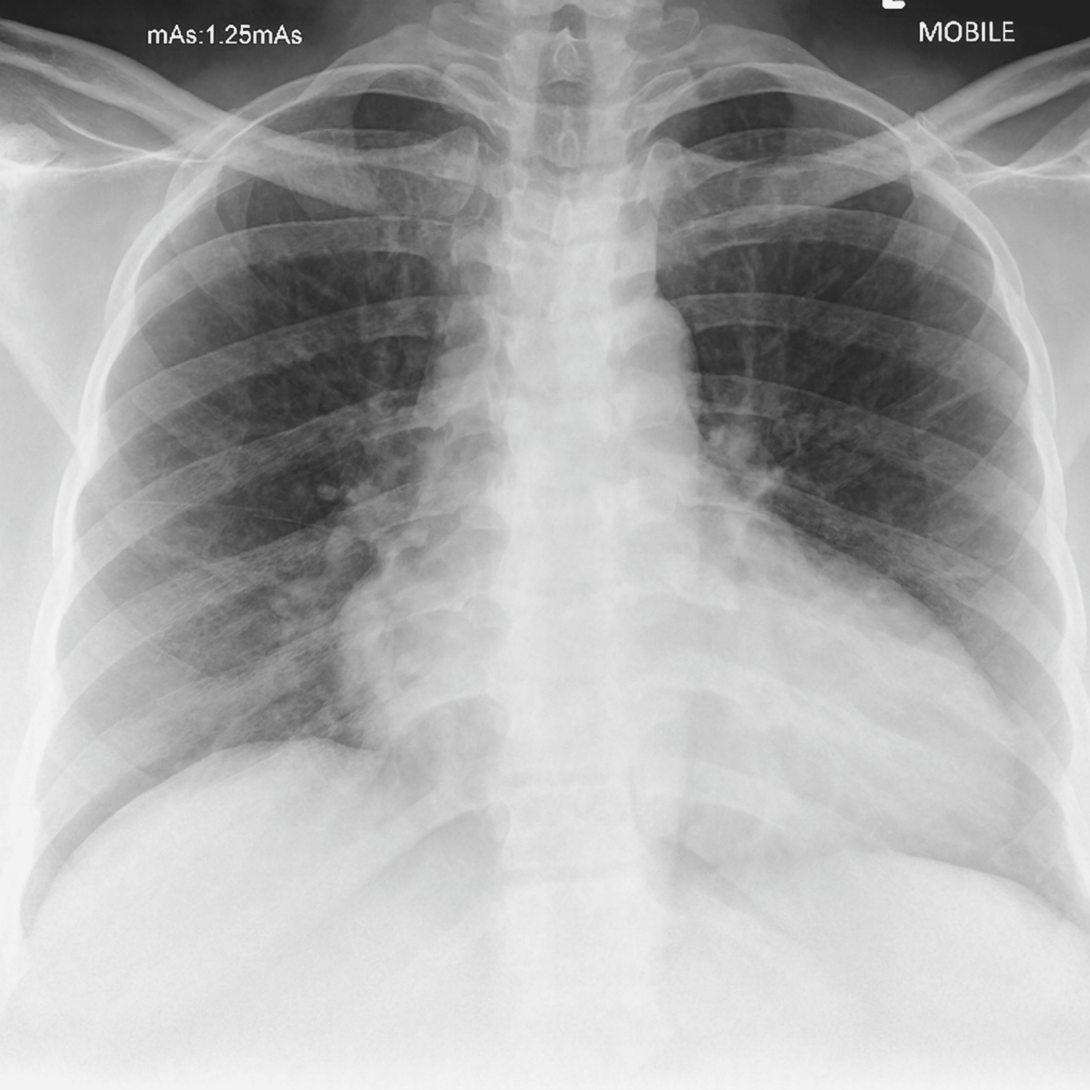
Chest X-ray showing pulmonary congestion with bilateral prominent vascular markings.

Initial investigations showed an iron-deficiency anaemia with haemoglobin confirmed as 96 g/L, platelets 194 × 10^9^/L, white cell count of 13.55 × 10^9^/L, and CRP 62 mg/L, and her clotting, liver, and renal function test results were normal, as summarised in Table 1. Viral swabs were negative for SARS-CoV-2, rhinovirus, enterovirus, adenovirus, respiratory syncytial virus (RSV), influenza A, and influenza B. Chest X-ray revealed pulmonary congestion (Figure 2).

**Table 1:** Laboratory results. ALT: Alanine aminotransferase; TBIL: Total bilirubin; PT: Prothrombin time; APTT: Activated partial thromboplastin time.

Investigation	Value	Reference range
Lactate	0.7 mmol/L	0.5-1.6 mmol/L
Haemoglobin	96 g/L	115-160 g/L
White cell count	13.55 × 10⁹/L	4.0-11.0 × 10⁹/L
Platelets	194 × 10⁹/L	150-450 × 10⁹/L
CRP	62 mg/L	0-5 mg/L
Sodium	134 mmol/L	133-146 mmol/L
Potassium	3.7 mmol/L	3.5-5.3 mmol/L
Creatinine	30 µmol/L	49-90 µmol/L (non-pregnancy range)
ALT	21 U/L	30-130 U/L
TBIL	14 µmol/L	0-21 µmol/L
PT	10.6 s	10.0-12.5 s
APTT	23.4 s	21.0-29.0 s

Diagnosis and treatment

The patient improved significantly after resuscitation, and foetal tachycardia normalised, resulting in criteria being met on CTG; however, she began to have recurring temperature spikes despite IV Tazocin. Apart from this, there was a high suspicion of meningitis based on her complaint of headache, photophobia, and findings of nuchal rigidity. We consulted with the microbiologist and medical team regarding optimisation of antibiotics, and it was advised to discontinue Tazocin and commence ceftriaxone and metronidazole as treatment for meningitis.

Twenty-four hours later, preliminary blood culture results indicated suspected Listeria. Amoxicillin was thus added to her antibiotic regimen. On day 3 of admission, there was confirmation of the blood culture results of Listeria, and so ceftriaxone and metronidazole were discontinued, and gentamicin (3 mg/kg daily) was added to the amoxicillin.

**Figure 3:**
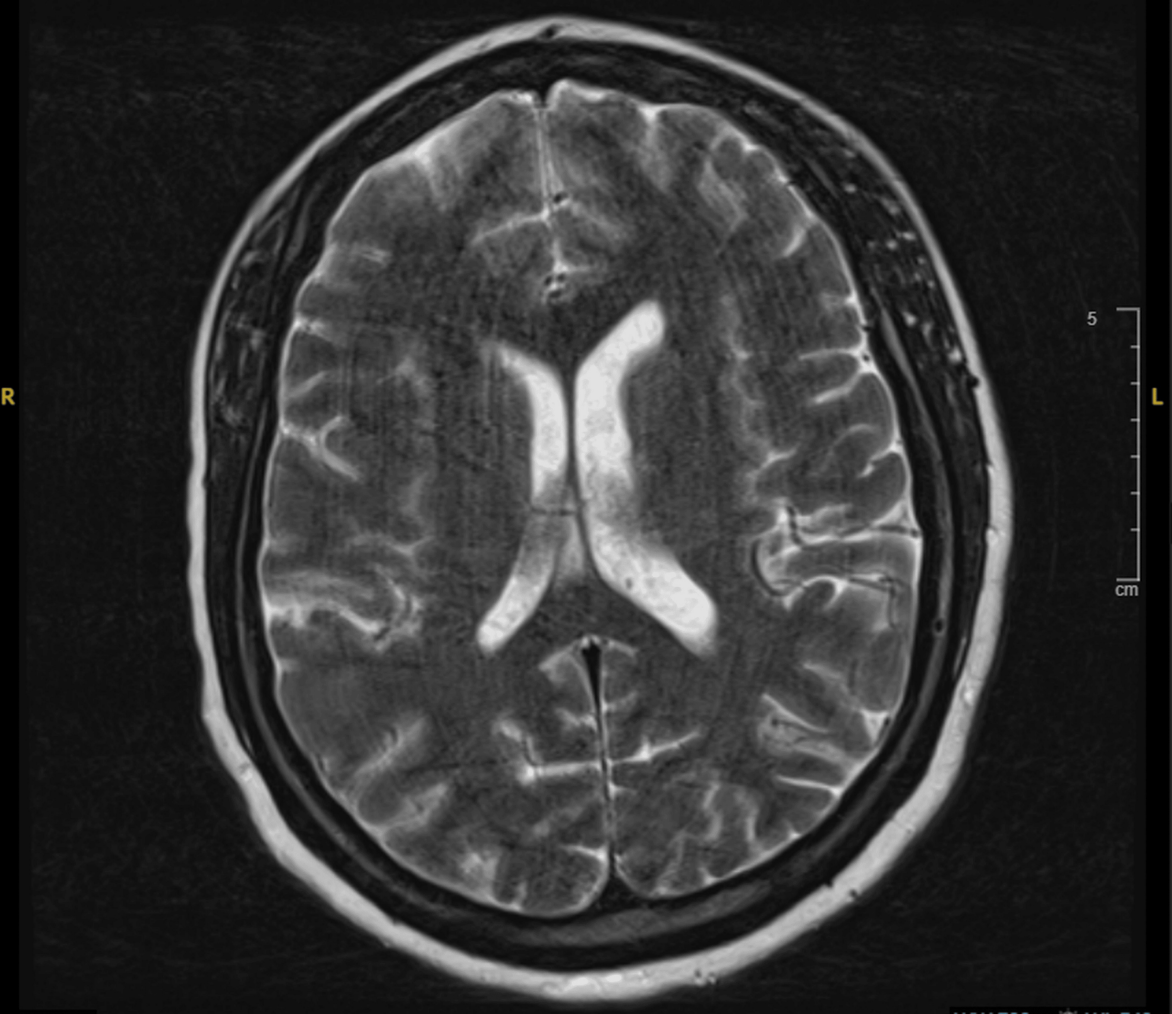
MRI brain showing no acute intracranial findings.

The patient’s recurring temperature spikes improved; however, it was thought that she had neurological sequelae, as she seemed to have poor memory retention, so concerns of cerebritis were raised, which can be due to Listeria. As such, she had a mental state examination, which was found to be normal, along with repeat blood cultures to determine the efficacy of the antibiotic regimen, and an MRI brain was requested. The MRI showed no acute intracranial findings and she continued to improve (Figure 3).

On day 6 of admission, she had a foetal growth scan, which showed an estimated foetal weight at the 95th percentile, with normal Dopplers and liquor volume. On day 7 of admission, she had another temperature spike; however, she was continued on the ongoing antibiotic regimen.

She completed 8 days of gentamicin and 14 days of amoxicillin. She was then discharged home clinically well, 15 days after her initial presentation, for daily benzylpenicillin (14.4 g) with a peripherally inserted central catheter (PICC) line to complete her outpatient antibiotic therapy. She remained well during her outpatient follow-up and had a repeat MRI that showed no signs of infection, so she went on to complete a further 8 doses of her antibiotic regimen as per the microbiology team.

Follow-up and outcome

The remainder of her antenatal care was uneventful, and she presented for regular follow-up with normal growth scans and good diabetic and blood pressure control. Her labour was induced at 39+1 weeks’ gestation; however, due to a pathological CTG, she had an emergency caesarean section and the baby was born well with no sequelae of Listeria. As she had chronic hepatitis B, the baby was given a hepatitis B vaccination, and mother and baby were discharged 48 hours after birth. The placenta was not sent for culture and histology in the absence of neonatal symptoms, a normal postpartum course, and institutional practice at the time.

## Discussion

Pregnancy-associated listeriosis is uncommon but carries disproportionate maternal-fetal risk. In the United States, FoodNet data estimate an incidence of around 4-5 per 100,000 pregnant women, with pregnancy-associated cases representing a meaningful share of all listeriosis [11,12]. In the United Kingdom, approximately 180 cases occur annually in recent years, of which about 16% are pregnancy-associated; crude population incidence is ~0.28 per 100,000 [13,14]. European surveillance similarly records small numbers of pregnancy-associated cases with substantial fetal loss [15]. Large outbreaks, such as the 2017-2018 epidemic in South Africa, highlight the potential for severe maternal-neonatal disease when exposure occurs [16].

Pregnant patients, neonates, older adults, and immunocompromised individuals are at greatest risk for invasive disease [4,6]. Additional host factors include diabetes mellitus, chronic liver disease (including viral hepatitis), chronic kidney disease, alcohol use disorder, iron overload or high-iron states, and immunosuppressive therapies; gastric hypochlorhydria and proton pump inhibitor use may facilitate gastric survival of Listeria [4,6]. Because manifestations are often nonspecific (fever, myalgia, malaise, GI upset), diagnosis is challenging. Neurological features such as headache and photophobia may suggest meningitis, but neuroimaging can be normal, as in this case [17]. Blood culture remains the cornerstone of diagnosis; timely sampling is critical to guide therapy [4,9].

Use of a structured sepsis bundle, rapid cultures, timely antibiotics (ideally within the first hour when septic shock is suspected), appropriate fluids, and escalation when indicated, improves outcomes [1]. Maternal therapy, ideally at least 24 hours before delivery, reduces neonatal disease severity [8].

Diagnosing Listeria meningitis (neurolisteriosis)

In listeriosis, sepsis can progress to meningitis, encephalitis (including rhombencephalitis), septic shock, and multi-organ dysfunction; neonatal sequelae include pneumonia, meningitis, and death [18-19]. The gestational age at the time of the infection influences fetal outcome. First-trimester infections carry the highest risk of fetal loss; outcomes are better in the third trimester, though preterm delivery and neonatal sepsis remain concerns [19].

In our case, presentation at 29+5 weeks, rapid diagnosis, pathogen-directed antibiotics, and multidisciplinary care likely prevented vertical transmission and neonatal listeriosis.

Diagnosis is typically confirmed by culture of blood or CSF. CSF bacterial load may be low, so blood cultures can be more sensitive; Gram stain is often negative. Where available, multiplex CSF PCR improves speed and sensitivity [20]. The organism may require ~36 hours to grow in culture; serological tests are not widely used in routine care [4].

Empiric adult meningitis regimens in at-risk patients, including pregnancy, should include ampicillin or amoxicillin to cover Listeria in addition to standard ceftriaxone ± vancomycin. For directed therapy, high-dose ampicillin or amoxicillin with gentamicin is recommended for severe CNS disease due to synergistic bactericidal activity. Typical durations are ≥21 days for meningitis and 4-6 weeks for brain abscess or rhombencephalitis; bacteraemia without CNS involvement is commonly treated for ~14 days. Gentamicin is usually limited to the first 5-10 days with toxicity monitoring. Alternatives for severe β-lactam allergy include trimethoprim-sulfamethoxazole; cephalosporins alone are ineffective; and dexamethasone is not recommended for Listeria meningitis.

MRI with contrast is the preferred imaging modality for neurolisteriosis; CT is reserved prior to lumbar puncture for patients with focal deficits, papilloedema, seizures, or markedly altered mental status [17]. If delivery occurs, the placenta should be sent for culture and histology, which can be more sensitive than maternal blood cultures in pregnancy-associated disease [8,9].

## Conclusions

Listeria monocytogenes should remain in the differential diagnosis of febrile illness in pregnancy. Because presentation is non-specific and cephalosporins lack activity against Listeria, early blood cultures and prompt transition to ampicillin/amoxicillin (with gentamicin when indicated) are essential. In this third-trimester case, guideline-concordant therapy and coordinated multidisciplinary care led to full maternal recovery and an uncomplicated neonatal outcome.
